# Digital Teaching in Medical Education: Scientific Literature Landscape Review

**DOI:** 10.2196/32747

**Published:** 2022-02-09

**Authors:** Andy Wai Kan Yeung, Emil D Parvanov, Mojca Hribersek, Fabian Eibensteiner, Elisabeth Klager, Maria Kletecka-Pulker, Bernhard Rössler, Karl Schebesta, Harald Willschke, Atanas G Atanasov, Eva Schaden

**Affiliations:** 1 Ludwig Boltzmann Institute for Digital Health and Patient Safety Medical University of Vienna Vienna Austria; 2 Oral and Maxillofacial Radiology, Applied Oral Sciences and Community Dental Care Faculty of Dentistry The University of Hong Kong Hong Kong China; 3 Department of Translational Stem Cell Biology Medical University of Varna Varna Bulgaria; 4 Division of Pediatric Nephrology and Gastroenterology, Department of Pediatrics and Adolescent Medicine Comprehensive Center for Pediatrics Medical University of Vienna Vienna Austria; 5 Institute for Ethics and Law in Medicine University of Vienna Vienna Austria; 6 Department of Anaesthesia, Intensive Care Medicine and Pain Medicine Medical University of Vienna Vienna Austria; 7 Academic Simulation Center Vienna Vienna Austria; 8 Institute of Genetics and Animal Biotechnology of the Polish Academy of Sciences Jastrzebiec Poland

**Keywords:** medical education, digital teaching, virtual reality, augmented reality, anatomy, basic life support, satisfaction, bibliometric, medicine, life support, online learning, literature, trend, citation

## Abstract

**Background:**

Digital teaching in medical education has grown in popularity in the recent years. However, to the best of our knowledge, no bibliometric report to date has been published that analyzes this important literature set to reveal prevailing topics and trends and their impacts reflected in citation counts.

**Objective:**

We used a bibliometric approach to unveil and evaluate the scientific literature on digital teaching research in medical education, demonstrating recurring research topics, productive authors, research organizations, countries, and journals. We further aimed to discuss some of the topics and findings reported by specific highly cited works.

**Methods:**

The Web of Science electronic database was searched to identify relevant papers on digital teaching research in medical education. Basic bibliographic data were obtained by the “Analyze” and “Create Citation Report” functions of the database. Complete bibliographic data were exported to VOSviewer for further analyses. Visualization maps were generated to display the recurring author keywords and terms mentioned in the titles and abstracts of the publications.

**Results:**

The analysis was based on data from 3978 papers that were identified. The literature received worldwide contributions with the most productive countries being the United States and United Kingdom. Reviews were significantly more cited, but the citations between open access vs non–open access papers did not significantly differ. Some themes were cited more often, reflected by terms such as virtual reality, innovation, trial, effectiveness, and anatomy. Different aspects in medical education were experimented for digital teaching, such as gross anatomy education, histology, complementary medicine, medicinal chemistry, and basic life support. Some studies have shown that digital teaching could increase learning satisfaction, knowledge gain, and even cost-effectiveness. More studies were conducted on trainees than on undergraduate students.

**Conclusions:**

Digital teaching in medical education is expected to flourish in the future, especially during this era of COVID-19 pandemic.

## Introduction

Rapid advancements in information technology and worldwide internet access potentially allow for the full substitution of traditional face-to-face medical education with digital teaching methods (including but not limited to remote teaching). Overall, digital teaching applications may be categorized as distance learning applications vs computer-assisted interaction [1]. In the early and mid-1980s, the very first online courses for undergraduate, postgraduate, and adult education were established, and even online degree programs were introduced [2]. With the public access to the World Wide Web granted by its developers in the early 1990s, digital teaching has become increasingly popular. Similar to traditional face-to-face teaching, digital teaching also needs to be approached from various perspectives, such as achieving competency in pedagogical, technological, and content knowledge [3]. To maintain a positive learning experience, the teaching environment should also account for social, cognitive, and teaching presence [4]. Digital teaching is considered challenging and often faces a rather high attrition rate in comparison to on-campus teaching due to various reasons, including technical difficulties, perceived isolation, content confusion, poor academic performance, and lack of motivation [5]. Digital teaching allows for more flexibility with work or family commitments [6,7] and reduces costs [8,9]. However, some studies have questioned the degree of improvement of student outcomes by remote learning [7,10,11]. In addition, although students experience digital learning as an entertaining new way to study, they do not consider it to replace classical didactic methods [12]. Often, digital teaching is used together with traditional approaches in so-called hybrid (blended) learning. Although it has received higher acceptance by students, blended learning did not exhibit a significant difference in comparison to the traditional methods based on final test scores [13,14]. Digital teaching in medical education shares similarities with other educational areas as it enhances self-directed learning and computer literacy skills. Yet it also follows its own specific aims, such as to enhance collaboration skills, problem solving skills, critical thinking, and filling the gap between theory and practice [15].

These teaching methods gained great importance during the COVID-19 pandemic, as remote teaching methods provided the opportunity to bypass the mitigation measurements (eg, social distancing). This is reflected by an enormous increase in online and remote schooling during the time of the pandemic [16,17]. In the context of medical education, digital teaching is applicable for teaching medical students, resident or specialty training, and continuing medical education of physicians. Available medical digital teaching platforms were primarily utilized by medical schools and consisted of video clips, virtual models, and so on. Positive aspects of these platforms are the possibility of regular updates, easy accessibility, and proven efficiency of knowledge transfer allowing self-directed learning [11]. Importantly, knowledge transfer is believed to be a key element of medical education, and success in this form of self-directed learning means being able to apply knowledge in a new context, which was being learned in another context beforehand [18]. The major potential barriers for digital teaching applications in medical education are several: the presence of technology or infrastructure (valid especially for low-income countries); institutional support; trained educators; and overall acceptance by the students.

Thousands of scientific studies have explored different kinds of digital teaching applications in medical education. In this work, we aimed to gain insights into the tendencies and features of this scientific area by the application of a total scale bibliometric analysis, an approach that has proven its value in the characterization of diverse scientific areas with medical significance [19-21]. We also aimed to identify the most productive entities and reveal recurring terms from the current literature on digital teaching in medical education.

## Methods

### Data Source and Search Strategy

On July 1, 2021, the digital Web of Science (WoS) core collection database was accessed and queried with the following search string: TOPIC: (“eTeaching*” OR “online teaching*” OR “E-teaching*” OR “electronic teaching*” OR “computer-assisted teaching*” OR “computer-mediated teaching*” OR “computer-based teaching*” OR “digital teaching*” OR “online course*” OR “eLearning*” OR “online learning*” OR “E-learning*” OR “electronic learning*” OR “computer-assisted learning*” OR “computer-mediated learning*” OR “computer-based learning*” OR “digital learning*”) AND TOPIC: (“medic*”). The query identified publications mentioning these words and their derivatives in the title, abstract and, keywords. The “Analyze” and “Create Citation Report” functions of WoS were deployed for initial analyses and frequency counting. The full records of the resultant publications were exported to VOSviewer, version 1.6.15 (Leiden University) for further bibliometric analyses. Normalized citations were computed in VOSviewer by considering the average number of citations received by the documents published in the same year and included in the data set (a score of >1 indicates higher-than-average citations compared with the documents published in the same year). As an exploratory analysis, we further analyzed publications with authors based on low-income economies according to the World Bank [22].

### Visualization of Scientific Landscapes

The VOSviewer [23] generated bubble maps that visualized the recurring terms and their citation per publication (CPP). Terms that appeared in at least 1% of the analyzed publications (n≥40) were visualized. Similarly, author keywords that appeared in at least 3 publications were visualized.

### Statistical Analysis

Two-sample *t* tests were conducted to analyze if the CPP showed a significant difference between original articles and reviews, and between open access (OA) and non-OA papers. Statistical tests were performed with SPSS, version 26.0 (IBM Corp). The results were deemed significant if *P*<.05.

## Results

### Overall Literature Landscape

**Figure 1:**
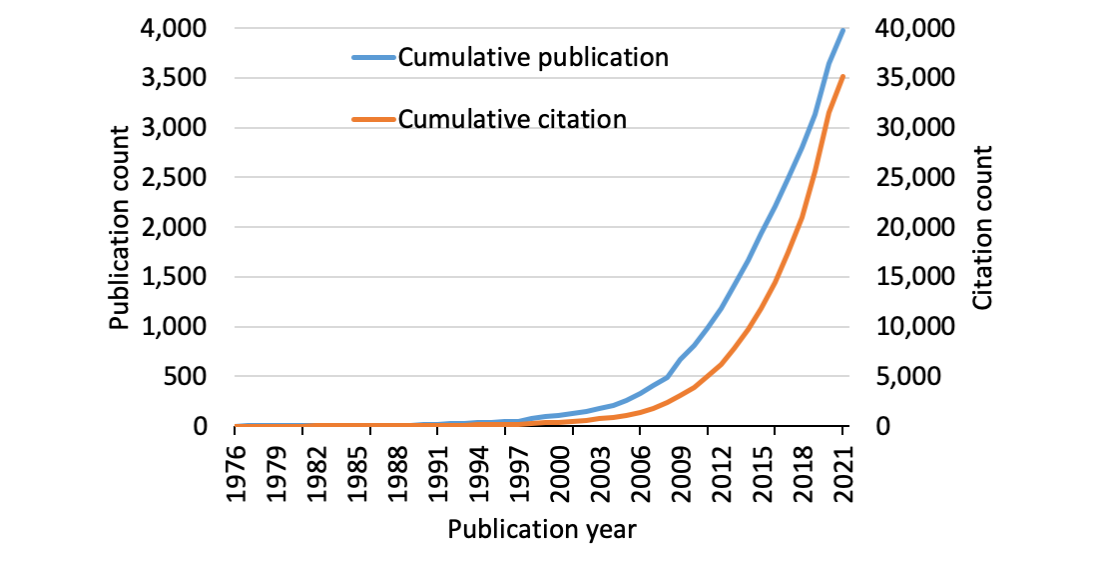
Cumulative publication and citation count of digital teaching in medical education.

Our literature search yielded a total of 3978 documents, which collectively accumulated 35,104 citations (Figure 1), reflecting a CPP of 8.82 and h-index of 65. The first paper on this topic was published in Lancet in 1976, reporting the experimentation of computer-assisted learning among 5th year medical students at Glasgow University [24]. The study reported that 79 out of 80 students were keen to have further such tuition. Meanwhile, in 2020, when the COVID-19 pandemic affected the whole world, the yearly publication count on digital teaching suddenly increased to 515, up from 200-300 in the prior 7 years. About 72.1% of the retrieved documents were original articles (n=2870, CPP=9.8), whereas 6.0% were reviews (n=239, CPP=21.0). The remaining were mainly proceedings papers, editorial materials, and meeting abstracts. Hence, the article-to-review ratio was 12:1. Less than half (41.5%) were OA (n=1649, CPP=9.3), whereas over half were non-OA (n=2329, CPP=8.5). Reviews were significantly more often cited (*P*<.001) than original articles. Moreover, the citations between OA vs non-OA papers did not significantly differ (*P*=.331).

The most productive authors, organizations, journals, and journal categories are listed in Table 1. The contributors were mostly from Europe and North America. The reports were mainly published in medical education journals.

**Figure 2:**
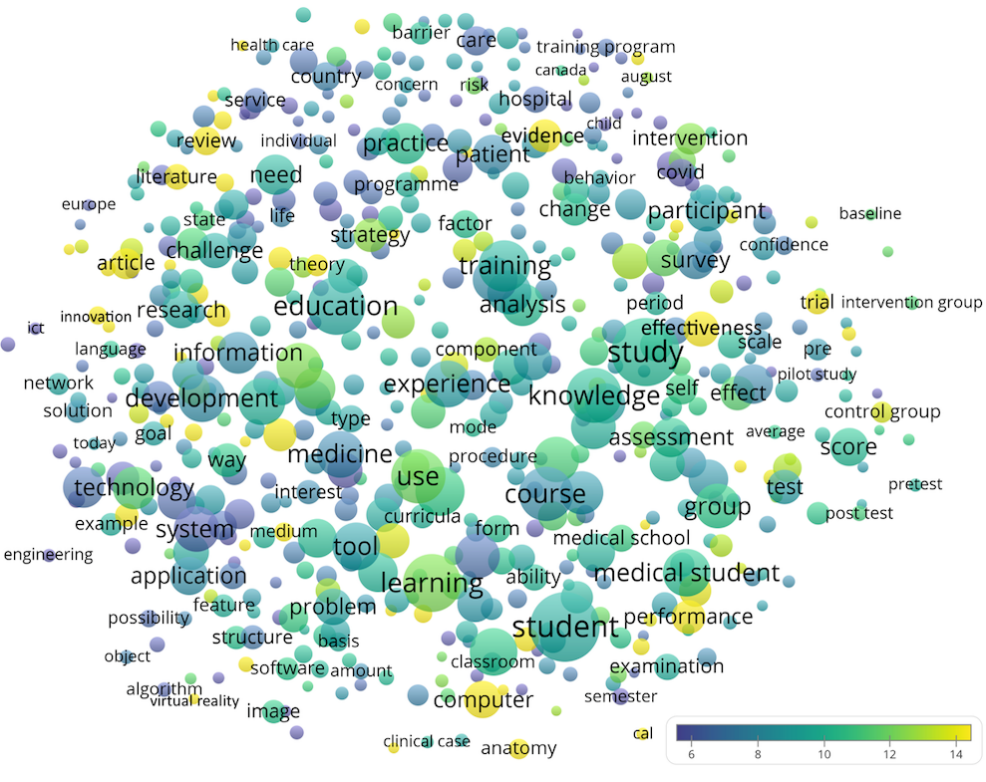
Term map showing recurring terms (n≥40) from the titles and abstracts of the analyzed papers. Bubble colors indicate citations per publication, their size indicates frequency count, and their proximity indicates the frequency of their coappearance.

**Figure 3:**
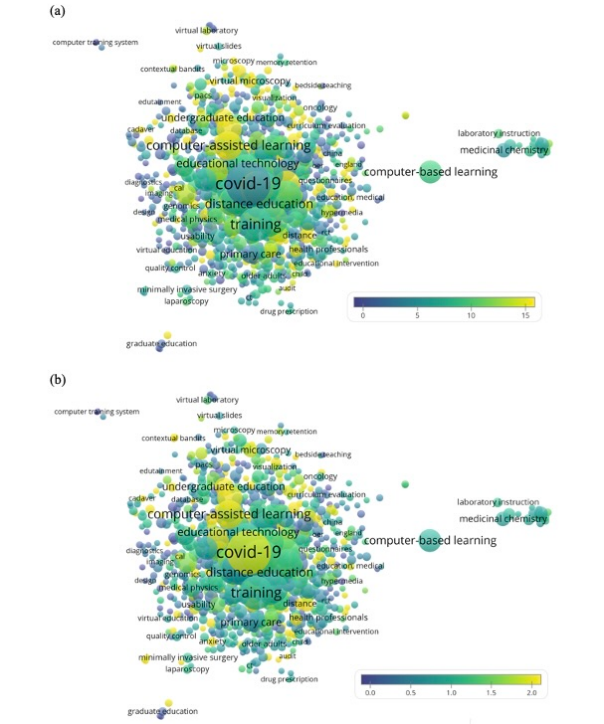
Term map showing recurring author keywords (n≥3) from the analyzed papers. Bubble color indicates (a) citations per publication and (b) average normalized citations (score of >1 indicates higher-than-average citations). Bubble sizes indicate frequency count and their proximity indicates the frequency of their coappearances.

The recurring terms in the titles and abstracts of the papers are depicted in Figure 2. Some themes were more highly cited (yellow bubbles), including general terms such as innovation (n=129 [3.2%], CPP=16.4), trial (n=220 [5.5%], CPP=14.3), effectiveness (n=474 [11.9%], CPP=14.8); terms describing modalities of digital teaching such as virtual reality (n=47 [1.2%], CPP=16.9), simulation (n=241 [6.1%], CPP=12.6), and massive open online course (MOOC), n=57 [1.4%], CPP=11.8); terms characterizing teaching disciplines such as anatomy (n=163 [4.1%], CPP=16.2), nursing (n=122 [3.1%], CPP=13.3), and surgery (n=129 [3.2%], CPP=8.8). It seemed that more studies were conducted on trainee (n=198 [5%], CPP=8.5) than undergraduate student (n=62 [1.6%], CPP=10.1). The recurring author keywords are depicted in Figure 3A (note that, for clarity, the following dominating keywords were omitted from the figure: e-learning [n=1010], medical education [n=500], education [n=352], online learning [n=240], blended learning [n=162], and elearning [n=108]). Different aspects in medical education were implied in digital teaching, such as gross anatomy education (n=50 [1.3%], CPP=31.0), histology (n=14 [0.4%], CPP=17.7), complementary medicine (n=6 [0.2%], CPP=3.0), medicinal chemistry (n=17 [0.4%], CPP=5.1), and basic life support (n=4 [0.1%], CPP=6.0). The term “COVID-19” had a rather low CPP. If we computed average normalized citations by normalizing the citations by the mean number of citations received by the documents published in the same year and included them in the data set, the recency yet importance of COVID-19 could be illustrated (normalized citation=1.95, where the citation rate achieved is equal to 1) (Figure 3B). Top 10 recurring keywords are listed in Table 2.

**Table 1:** Top 5 most productive authors, organizations, countries, journals, and journal categories.

Categories and subitems				CPP^a^
**Author, n (%)**				
	Martin R Fischer	27 (0.7)	21.3	
	David A Cook	18 (0.5)	55.9	
	Kieran Walsh	18 (0.5)	7.4	
	John Sandars	14 (0.4)	19.5	
	Nabil Zary	13 (0.3)	6.5	
**Organization, n (%)**				
	University of London	91 (2.3)	13.6	
	University of Toronto	86 (2.2)	12.9	
	Harvard University	77 (1.9)	9.1	
	University of California System	69 (1.7)	10.8	
	University of Munich	61 (1.5)	14.1	
**Country, n (%)**				
	The United States	991 (24.9)	12.0	
	The United Kingdom	558 (14.0)	13.1	
	Germany	434 (10.9)	7.3	
	Canada	310 (7.8)	13.4	
	Australia	237 (6.0)	11.0	
**Journal, n (%)**				
	BMC Medical Education	158 (4.0)	12.4	
	Medical Teacher	118 (3.0)	22.7	
	EDULEARN Proceedings	76 (1.9)	0.5	
	Anatomical Sciences Education	73 (1.8)	26.6	
	Studies in Health Technology and Informatics	54 (1.4)	3.1	
**Journal category, n (%)**				
	Education, scientific disciplines	910 (22.9)	14.5	
	Education, educational research	757 (19.0)	5.7	
	Health care sciences services	554 (13.9)	17.6	
	General internal medicine	351 (8.8)	8.2	
	Medical informatics	210 (5.3)	10.6	

^a^CPP: citation per publication.

**Table 2:** Top 10 recurring author keywords from the entire data set and from the low-income country publications.

Entire data set	n (%)	CPP^a^	Low-income country publications	n (%)	CPP
COVID-19	156 (3.9)	3.0	E-learning	9 (0.2)	23.8
Medical students	100 (2.5)	7.5	Medical education	4 (0.1)	45.3
Training	91 (2.3)	7.1	Training	3 (0.1)	2.7
Internet	87 (2.2)	14.7	Challenges	2 (0.1)	5.0
Teaching	82 (2.1)	9.2	COVID-19	2 (0.1)	4.5
Learning	73 (1.8)	7.0	Malawi	2 (0.1)	3.5
Evaluation	70 (1.8)	7.3	Research capacity strengthening	2 (0.1)	5.0
Continuing medical education	68 (1.7)	9.8	Undergraduate	2 (0.1)	2
Simulation	68 (1.7)	9.1	Low- and middle-income countries	1 (0.1)	170
Computer-assisted learning	66 (1.7)	26.5	Resource constrained	1 (0.1)	170

^a^CPP: citation per publication.

For completeness, Table 3 lists the top 10 most cited papers based on total and yearly citation count, respectively. Ranking by yearly citation count showed that many of the top 10 papers concerned COVID-19.

**Table 3:** Top 10 most cited papers based on total and yearly citation counts.

Paper		Reference	Total citations	Yearly citations
**Top 10 by total citations**				
	Ruiz JG, Mintzer MJ, Leipzig RM. The impact of E-learning in medical education.	[11]	982	61.4
	Ellaway R, Masters K. AMEE Guide 32: e-Learning in medical education Part 1: Learning, teaching and assessment.	[25]	298	21.3
	Greenhalgh T. Computer assisted learning in undergraduate medical education.	[26]	220	10.5
	Childs et al. Effective e‐learning for health professionals and students—barriers and their solutions. A systematic review of the literature—findings from the HeXL project.	[27]	205	12.1
	Cook DA. Web-based learning: pros, cons and controversies.	[28]	201	13.4
	Cook DA. The research we still are not doing: an agenda for the study of computer-based learning.	[29]	184	10.8
	Hamilton R. Nurses’ knowledge and skill retention following cardiopulmonary resuscitation training: a review of the literature.	[30]	174	10.2
	Frehywot et al. E-learning in medical education in resource constrained low-and middle-income countries.	[31]	170	18.9
	Liu Q et al. The effectiveness of blended learning in health professions: systematic review and meta-analysis.	[32]	161	26.8
	Mehta et al. Just imagine: new paradigms for medical education.	[33]	158	17.6
**Top 10 by yearly citations**				
	Ruiz JG, Mintzer MJ, Leipzig RM. The impact of E-learning in medical education.	[11]	982	61.4
	Thai NTT, De Wever B, Valcke M. The impact of a flipped classroom design on learning performance in higher education: Looking for the best “blend” of lectures and guiding questions with feedback.	[34]	138	27.6
	Liu et al. The effectiveness of blended learning in health professions: systematic review and meta-analysis.	[32]	161	26.8
	O’Doherty et al. Barriers and solutions to online learning in medical education–an integrative review.	[35]	102	25.5
	Aristovnik et al. Impacts of the COVID-19 pandemic on life of higher education students: A global perspective.	[36]	44	22.0
	Ellaway R, Masters K. AMEE Guide 32: e-Learning in medical education Part 1: Learning, teaching and assessment.	[25]	298	21.3
	Mukhtar et al. Advantages, Limitations and Recommendations for online learning during COVID-19 pandemic era. Pakistan journal of medical sciences.	[37]	41	20.5
	Sandhu P, de Wolf M. The impact of COVID-19 on the undergraduate medical curriculum. Med Educ Online.	[38]	39	19.5
	Schneider SL, Council ML. Distance learning in the era of COVID-19. Archives of dermatological research.	[39]	19	19.0
	Frehywot et al. E-learning in medical education in resource constrained low-and middle-income countries.	[31]	170	18.9
	Pei L, Wu H. Does online learning work better than offline learning in undergraduate medical education? A systematic review and meta-analysis.	[40]	54	18.0

By examining the data, we noticed that one of the keywords with highest CPP was “low- and middle-income countries” (n=3 [0.1%], CPP=57.7), listed by 2 original articles and 1 review [31,41,42]. Hence, we searched for this phrase in the entire data set (not limited to author keywords) and identified 19 original articles (CPP=6.9) and 7 reviews (CPP=44.1). These numbers suggested that the original research works on this aspect were not highly cited. For instance, the most cited work was a survey among students, residents, and lecturers in a medical faculty in Cameroon (38 citations) [43]. This work found that 84% of students and 58% of residents never had access to digital teaching resources but viewed digital teaching positively and wished to have it in their school. Thus, a high need for digital resources for medical teaching exists, at least in some parts of the world. To address such needs, the University of Dundee and the British Society for Antimicrobial Chemotherapy developed a MOOC on microbiology to cater education need in low- and middle-income countries, and found that 13% of participants were from Africa, 16% from Asia, 8% from Australia, 49% from Europe, 9% from North America, and 5% from South America [44].

**Figure 4:**
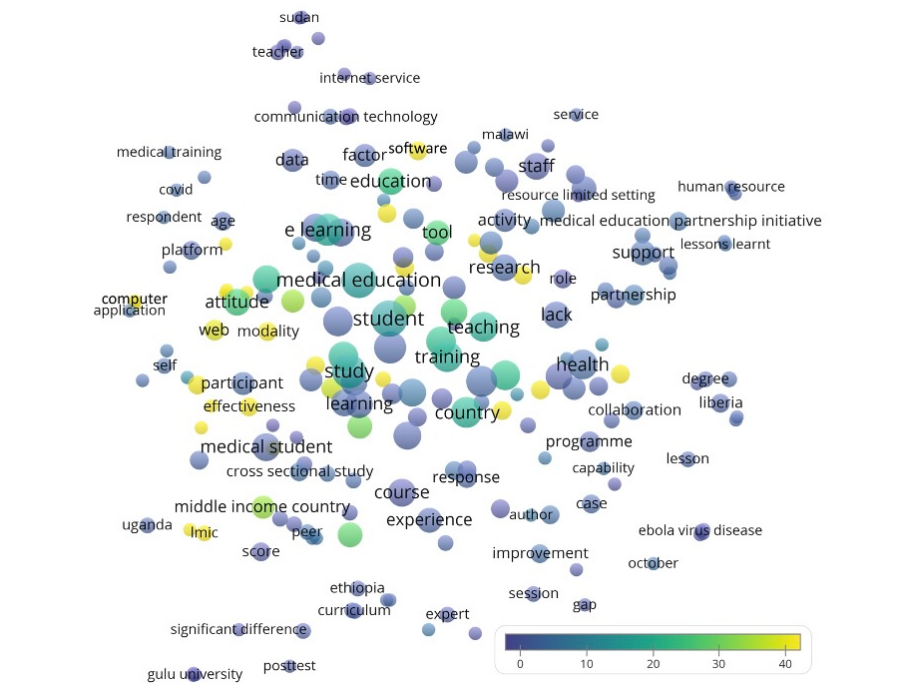
Term map showing recurring terms from the titles and abstracts of the papers from low-income economies. Bubble colors indicate citations per publication, their size indicates frequency count, and their proximity indicates the frequency of their coappearance.

Regarding publications with authors based in low-income economies according to the World Bank [22], we were able to identify a total of 29 publications from low-income economies with 262 citations, a CPP of 9.0, and an h-index of 7. A publication was included if it had an author based in low-income economies, irrespective of their position in the author list. The first document was published in 2009, an editorial that introduced a web-based learning environment by Omdurman Islamic University in Sudan [45]. The United States was involved in 8 (27%) of these 29 papers, whereas Sudan (n=7 [24%]), Ethiopia (n=6 [21%]), and Uganda (n=5 [17%]) were the most productive low-income countries. The most productive organization was Addis Ababa University in Ethiopia (n=5 [17%]), and the most productive journal was BMC Medical Education (n=3 [10%]). Figure 4 shows the recurring terms in the titles and abstracts (n≥2) from these publications. Terms reflected more basic concepts, such as resource-limited setting (n=2 [7%], CPP=0), computer (n=2 [7%], CPP=89.0), and medical education partnership initiative (n=4 [14%], CPP=5.0). Meanwhile, Table 2 shows that COVID-19 and training were recurring keywords shared by these papers and the entire data set.

## Discussion

### Major Findings

This bibliometric analysis of 3978 publications on digital education research in medicine revealed that this field began to grow rapidly in terms of both publication and citation counts in the 2000s. Original articles accounted for 72.1% of the literature. The article-to-review ratio was 12:1. This ratio was higher than that for literature sets of virtual reality application in medicine (5.9:1) [21] and medical errors (8.1:1) [19]. This implied that researchers working in digital teaching in medical education tended to conduct original research and find novelty instead of summarizing evidence from existing literature. The literature had heavy contributions from North America, Europe, and Oceania. Low-income countries together accounted for 0.9% of the publications, and their works were infrequently cited. This situation was similar in the emergency medicine literature, for which low-income countries published only 0.1% of all articles [46]. The small contribution by low-income countries was also identified in cardiovascular [47] and anesthesia literature [48].

With the current COVID-19 pandemic, digital teaching could prove itself very beneficial for medical education. As a reflection of these benefits and the wide application of digital education during the pandemic, COVID-19 was the most frequently occurring author keyword in the analyzed literature data set (Table 2). During the COVID-19 pandemic, many reports were published to share the perspectives as well as the impact and challenges of a sudden switch to digital teaching in the local settings, such as in Malaysia [49], Jordan [50], and Saudi Arabia [51]. Importantly, not all populations can readily access the internet for digital teaching. In Jordan, a survey on 652 medical students found that the overall satisfaction rate in medical digital teaching was only 26.8%, because 69.1% of them faced a main challenge of internet streaming quality and coverage [50]. Based on a focus group interview of 60 students, medical students in Saudi Arabia also faced some internet connection and synchronicity issues, but digital teaching was well accepted overall [51]. In Europe, poor internet connection was encountered by only 21.5% of 2721 surveyed medical students [52]. Although digital teaching can take place in many formats, internet accessibility remains to be a mandatory requirement. In countries and regions where medical students cannot connect to the internet anytime and anywhere, perhaps asynchronous formats will be more suitable, such as a MOOC course. A MOOC course that teaches emergency medical practice may deliver the teaching with good student satisfaction and, at the same time, effectively reduce the cross-infection risk between teachers, clinical staff, medical students, and patients [53].

The following discussion covers the principal findings from the most cited original articles in the literature set. For instance, in the setting of problem-based learning (PBL), a blended version was found to be as effective as the traditional face-to-face approach in terms of test results; it was also significantly superior in terms of subjective learning gains and satisfaction with easy revisits to the web-based learning modules [54]. A similar study on learning acid-base physiology found that students not only had higher satisfaction regarding the web-based PBL compared with the traditional PBL, but also a significantly higher test score with a medium effect size [55]. Performance enhancement was similarly observed for basic life support learning with web-based virtual patient encounters over standard training [56]. Regarding the web tools, it was advocated that YouTube (YouTube LLC) could be a very useful platform to store and disseminate tailor-made teaching videos such as those dedicated to human anatomy [57]. Moreover, a learning period as short as 30 minutes with a mobile phone with augmented reality blended learning environment could already bring about a greater knowledge gain than a traditional textbook [58]. Another benefit of digital teaching was cost-effectiveness: it was estimated that blended learning could cut costs by 24% compared to the traditional face-to-face approach [59]. However, initial costs of creation and preparation of digital teaching tools, including provision of an adequate information technology environment, may exceed those of traditional face-to-face approaches and may therefore act as a possible barrier to implementation.

Taken together, this short overview of the most cited original articles in the analyzed literature set is illustrative of the diversity of digital tools that could be used for medical education and the benefits that they are offering.

To the authors’ knowledge, no previous bibliometric analysis on digital teaching research in the medical literature has been published. A recent study on 10 e-learning journals found that the field shifted its foci from online learning, distance education, and pedagogical issues to mobile learning and interactive learning environments [60]. Meanwhile, research on e-learning in higher education was predominated by Spain and published in EDULEARN Proceedings [61]. These entities ranked 6th and 3rd in our predefined literature set, as listed in Table 1. By contrast, current results were consistent to a general e-learning literature analysis that identified the United States and the United Kingdom as the most productive countries; however, in both countries, chemical and engineering journals predominated instead of medical education ones [62,63]. Further, it was found that e-learning literature could be clustered into 7 foci: social sciences, psychology, medicine, health professions, life sciences, physical sciences or engineering, and computer science [64]. Moreover, it seemed that the contribution of computer science was on the decline whereas that of social sciences was gradually increasing in the scientific literature on digital education [65]. Finally, the current results were similar to that of the health sciences literature, where BMC Medical Education and Medical Teacher were the top 2 most productive journals, and COVID-19 was one of the most frequently mentioned keywords [66].

### Limitations

Some papers might not be indexed by WoS and may thus be missed from the analysis presented in this study. Besides, it was not possible to assess the methodology and reporting quality of each experimental study due to the large number of publications involved. Moreover, the contributions from the low-income countries might be underestimated, as their works might be published in local journals or journals not indexed by WoS. This limitation could be partially addressed by extending the search to other databases such as Scopus and Education in Africa, hosted by AfricaBib. However, different databases count publication and citation data differently, which hinders merging such data for the kind of bibliometric analysis applied in our work. Nevertheless, readers should be aware that searching other databases with the same search string is expected to result in additional relevant publications (eg, identical PubMed search identified 5383 papers as compared with the 3978 papers identified in WoS, which was analyzed in this study) since WoS has more stringent requirements for indexing, requiring more time to achieve indexing for new journals. However, with respect to the latter consideration, it is a reasonable assumption that the most significant and impactful scientific works would be more often published in established journals already indexed in WoS (on average, the WoS data set analyzed by us consist of studies with higher significance and impact, and this is not necessarily a limitation; rather, it can also be seen as a kind of filtering that excludes papers from less established journals). Along this line of thought, we should however emphasize that “less established journals” would not necessarily imply inferior journal quality; while other databases sometimes index journals that simply do not meet the stringent quality criteria of WoS, at the same time, there are examples of newly emerging journals of high quality, which are well on their way to being indexed in WoS. One highly relevant example for this area of research would be JMIR Medical Education, edited by Nabil Zary, who was one of the most productive researchers identified in our study (Table 1). Furthermore, it should be noticed that the CPP data were based on all publication types, not just original articles. Therefore, a high CPP reflected in our study does not necessarily mean that only original research articles concerning certain terms were highly cited; this parameter is also influenced by the citation rates of relevant reviews, proceedings papers, editorial material, and meeting abstracts. Readers should be aware of these limitations when interpreting the results. Moreover, considering that Scopus and Google Scholar are becoming increasingly used for the assessment of academic performance in different environments (often in low- and middle-income countries), future studies assessing the publication practices based on these databases are expected to gain further insights.

### Conclusions

The analyzed literature in the field of digital teaching research in medicine contained 3978 publications. The literature received worldwide contributions with the most productive countries being the United States and the United Kingdom. Reviews were significantly more cited, but the citations between OA vs non-OA papers did not significantly differ. Some themes were more highly cited, such as virtual reality, innovation, trial, effectiveness, and anatomy. Different aspects in medical education were experimented for digital teaching, such as gross anatomy education, histology, complementary medicine, medicinal chemistry, and basic life support. Some studies have shown that digital teaching could increase learning satisfaction, knowledge gain, and even cost-effectiveness. Digital teaching in medical education is expected to flourish in the future, especially in light of the COVID-19 pandemic occurrence, which brought the advantages of the digital education approach to the spotlight. This would be particularly useful for clinical teaching during pandemics, gaining insights into highly infectious diseases or rare diseases that do not have available cases in a local setting.

## Abbreviations

CPP: citation per publication
MOOC: massive open online course
OA: open access
PBL: problem-based learning
WoS: Web of Science

## References

[1] Moore JL, Dickson-Deane C, Galyen K (2011). e-Learning, online learning, and distance learning environments: Are they the same?. The Internet and Higher Education.

[2] Harasim L (2000). Shift happens: online education as a new paradigm in learning. The Internet and Higher Education.

[3] Mishra P, Koehler MJ (2006). Technological Pedagogical Content Knowledge: A Framework for Teacher Knowledge. Teachers College Rec.

[4] Garrison D, Anderson T, Archer W (1999). Critical Inquiry in a Text-Based Environment: Computer Conferencing in Higher Education. The Internet and Higher Education.

[5] Roddy C, Amiet D, Chung J, Holt C, Shaw L, McKenzie S, Garivaldis F, Lodge Jm, Mundy Me (2017). Applying Best Practice Online Learning, Teaching, and Support to Intensive Online Environments: An Integrative Review. Front. Educ.

[6] Johnson H, Cuellar Mejia M (2014). Online Learning and Student Outcomes in Community Colleges. Public Policy Institute of California.

[7] Xu D, Xu Y (2019). The promises and limits of online higher education: understanding how distance education affects access, cost, and quality.

[8] Bettinger EP, Fox L, Loeb S, Taylor ES (2017). Virtual Classrooms: How Online College Courses Affect Student Success. American Economic Review.

[9] Deming DJ, Goldin C, Katz LF, Yuchtman N (2015). Can Online Learning Bend the Higher Education Cost Curve?. American Economic Review.

[10] Hadadgar A, Changiz T, Masiello I, Dehghani Z, Mirshahzadeh N, Zary N (2016). Applicability of the theory of planned behavior in explaining the general practitioners eLearning use in continuing medical education. BMC Med Educ.

[11] Ruiz JG, Mintzer MJ, Leipzig RM (2006). The impact of E-learning in medical education. Acad Med.

[12] Warnecke E, Pearson S (2011). Medical students' perceptions of using e-learning to enhance the acquisition of consulting skills. Australas Med J.

[13] Blissitt AM (2016). Blended Learning Versus Traditional Lecture in Introductory Nursing Pathophysiology Courses. J Nurs Educ.

[14] Sadeghi R, Sedaghat MM, Sha Ahmadi F (2014). Comparison of the effect of lecture and blended teaching methods on students' learning and satisfaction. J Adv Med Educ Prof.

[15] Chornyi V, Vakulych M (2020). Specificities of Remote Teaching of Traumatology and Orthopedics Course to Medical Students. RREM.

[16] Bacher-Hicks A, Goodman J, Mulhern C (2020). Inequality in Household Adaptation to Schooling Shocks: Covid-Induced Online Learning Engagement in Real Time. National Bureau of Economic Research.

[17] Hamilton LS, Grant D, Kaufman JH, Diliberti MK, Schwartz HL, Hunter GP, Setodji CM, Young CJ (2020). COVID-19 and the state of K-12 schools: results and technical documentation from the Spring 2020. American Educator Panels COVID-19 surveys. RAND Corporation.

[18] Kuipers DA, Terlouw G, Wartena BO, van 't Veer JT, Prins JT, Pierie JPE (2017). The Role of Transfer in Designing Games and Simulations for Health: Systematic Review. JMIR Serious Games.

[19] Atanasov AG, Yeung AWK, Klager E, Eibensteiner F, Schaden E, Kletecka-Pulker M, Willschke H (2020). First, Do No Harm (Gone Wrong): Total-Scale Analysis of Medical Errors Scientific Literature. Front Public Health.

[20] Yeung AWK, Atanasov AG, Sheridan H, Klager E, Eibensteiner F, Völkl-Kernsock S, Kletecka-Pulker M, Willschke H, Schaden E (2020). Open Innovation in Medical and Pharmaceutical Research: A Literature Landscape Analysis. Front Pharmacol.

[21] Yeung AWK, Tosevska A, Klager E, Eibensteiner F, Laxar D, Stoyanov J, Glisic M, Zeiner S, Kulnik ST, Crutzen R, Kimberger O, Kletecka-Pulker M, Atanasov AG, Willschke H (2021). Virtual and Augmented Reality Applications in Medicine: Analysis of the Scientific Literature. J Med Internet Res.

[22] (2021). World Bank Country and Lending Groups. The World Bank.

[23] van Eck NJ, Waltman L (2010). Software survey: VOSviewer, a computer program for bibliometric mapping. Scientometrics.

[24] Murray T, Barber J, Cupples R, Hannay D, Scott D (1976). Computer-assisted learning in undergraduate medical teaching. The Lancet.

[25] Ellaway R, Masters K (2008). AMEE Guide 32: e-Learning in medical education Part 1: Learning, teaching and assessment. Med Teach.

[26] Greenhalgh T (2001). Computer assisted learning in undergraduate medical education. BMJ.

[27] Childs S, Blenkinsopp E, Hall A, Walton G (2005). Effective e-learning for health professionals and students--barriers and their solutions. A systematic review of the literature--findings from the HeXL project. Health Info Libr J.

[28] Cook DA (2007). Web-based learning: pros, cons and controversies. Clin Med (Lond).

[29] Cook David A (2005). The research we still are not doing: an agenda for the study of computer-based learning. Acad Med.

[30] Hamilton R (2005). Nurses' knowledge and skill retention following cardiopulmonary resuscitation training: a review of the literature. J Adv Nurs.

[31] Frehywot S, Vovides Y, Talib Z, Mikhail N, Ross H, Wohltjen H, Bedada S, Korhumel K, Koumare AK, Scott J (2013). E-learning in medical education in resource constrained low- and middle-income countries. Hum Resour Health.

[32] Liu Q, Peng W, Zhang F, Hu R, Li Y, Yan W (2016). The Effectiveness of Blended Learning in Health Professions: Systematic Review and Meta-Analysis. J Med Internet Res.

[33] Mehta NB, Hull AL, Young JB, Stoller JK (2013). Just Imagine. Academic Medicine.

[34] Thai NTT, De Wever B, Valcke M (2017). The impact of a flipped classroom design on learning performance in higher education: Looking for the best “blend” of lectures and guiding questions with feedback. Computers & Education.

[35] O'Doherty Diane, Dromey M, Lougheed J, Hannigan A, Last J, McGrath D (2018). Barriers and solutions to online learning in medical education - an integrative review. BMC Med Educ.

[36] Aristovnik A, Keržič D, Ravšelj D, Tomaževič N, Umek L (2020). Impacts of the COVID-19 Pandemic on Life of Higher Education Students: A Global Perspective. Sustainability.

[37] Mukhtar K, Javed K, Arooj M, Sethi A (2020). Advantages, Limitations and Recommendations for online learning during COVID-19 pandemic era. Pak J Med Sci.

[38] Sandhu P, de Wolf M (2020). The impact of COVID-19 on the undergraduate medical curriculum. Med Educ Online.

[39] Schneider SL, Council ML (2021). Distance learning in the era of COVID-19. Arch Dermatol Res.

[40] Pei L, Wu H (2019). Does online learning work better than offline learning in undergraduate medical education? A systematic review and meta-analysis. Med Educ Online.

[41] Bediang G, Perrin C, Ruiz de Castañeda R, Kamga Y, Sawadogo A, Bagayoko CO, Geissbuhler A (2014). The RAFT Telemedicine Network: Lessons Learnt and Perspectives from a Decade of Educational and Clinical Services in Low- and Middle-Incomes Countries. Front Public Health.

[42] Vaca SD, Warstadt NM, Ngayomela IH, Nungu R, Kowero ES, Srivastava S (2018). Evaluation of an E-Learning Course for Clubfoot Treatment in Tanzania: A Multicenter Study. J Med Educ Curric Dev.

[43] Bediang G, Stoll B, Geissbuhler A, Klohn AM, Stuckelberger A, Nko'o Samuel, Chastonay P (2013). Computer literacy and E-learning perception in Cameroon: the case of Yaounde Faculty of Medicine and Biomedical Sciences. BMC Med Educ.

[44] Sneddon J, Barlow G, Bradley S, Brink A, Chandy S, Nathwani D (2018). Development and impact of a massive open online course (MOOC) for antimicrobial stewardship. J Antimicrob Chemother.

[45] Hussain A (2009). e-Learning: The nextbig thing in medical education. Sud Jnl Med Sci.

[46] Li Q, Jiang Y, Zhang M (2012). National representation in the emergency medicine literature: a bibliometric analysis of highly cited journals. Am J Emerg Med.

[47] Huffman MD, Baldridge A, Bloomfield GS, Colantonio LD, Prabhakaran P, Ajay VS, Suh S, Lewison G, Prabhakaran D (2013). Global cardiovascular research output, citations, and collaborations: a time-trend, bibliometric analysis (1999-2008). PLoS One.

[48] Bould M, Boet S, Riem N, Kasanda C, Sossou A, Bruppacher H (2010). National representation in the anaesthesia literature: a bibliometric analysis of highly cited anaesthesia journals. Anaesthesia.

[49] Sundarasen S, Chinna K, Kamaludin K, Nurunnabi M, Baloch GM, Khoshaim HB, Hossain SFA, Sukayt A (2020). Psychological Impact of COVID-19 and Lockdown among University Students in Malaysia: Implications and Policy Recommendations. Int J Environ Res Public Health.

[50] Al-Balas M, Al-Balas HI, Jaber HM, Obeidat K, Al-Balas H, Aborajooh EA, Al-Taher R, Al-Balas B (2020). Distance learning in clinical medical education amid COVID-19 pandemic in Jordan: current situation, challenges, and perspectives. BMC Med Educ.

[51] Khalil R, Mansour AE, Fadda WA, Almisnid K, Aldamegh M, Al-Nafeesah A, Alkhalifah A, Al-Wutayd O (2020). The sudden transition to synchronized online learning during the COVID-19 pandemic in Saudi Arabia: a qualitative study exploring medical students' perspectives. BMC Med Educ.

[52] Dost S, Hossain A, Shehab M, Abdelwahed A, Al-Nusair L (2020). Perceptions of medical students towards online teaching during the COVID-19 pandemic: a national cross-sectional survey of 2721 UK medical students. BMJ Open.

[53] Zhou T, Huang S, Cheng J, Xiao Y (2020). The Distance Teaching Practice of Combined Mode of Massive Open Online Course Micro-Video for Interns in Emergency Department During the COVID-19 Epidemic Period. Telemed J E Health.

[54] Woltering V, Herrler A, Spitzer K, Spreckelsen C (2009). Blended learning positively affects students' satisfaction and the role of the tutor in the problem-based learning process: results of a mixed-method evaluation. Adv Health Sci Educ Theory Pract.

[55] Taradi SK, Taradi M, Radic Kresimir, Pokrajac N (2005). Blending problem-based learning with Web technology positively impacts student learning outcomes in acid-base physiology. Adv Physiol Educ.

[56] Lehmann R, Thiessen C, Frick B, Bosse HM, Nikendei C, Hoffmann GF, Tönshoff Burkhard, Huwendiek S (2015). Improving Pediatric Basic Life Support Performance Through Blended Learning With Web-Based Virtual Patients: Randomized Controlled Trial. J Med Internet Res.

[57] Jaffar AA (2012). YouTube: An emerging tool in anatomy education. Anat Sci Educ.

[58] Albrecht U, Folta-Schoofs K, Behrends M, von Jan U (2013). Effects of mobile augmented reality learning compared to textbook learning on medical students: randomized controlled pilot study. J Med Internet Res.

[59] Maloney S, Nicklen P, Rivers G, Foo J, Ooi YY, Reeves S, Walsh K, Ilic D (2015). A Cost-Effectiveness Analysis of Blended Versus Face-to-Face Delivery of Evidence-Based Medicine to Medical Students. J Med Internet Res.

[60] Bai Y, Li H, Liu Y (2020). Visualizing research trends and research theme evolution in E-learning field: 1999–2018. Scientometrics.

[61] López-Belmonte Jesús, Segura-Robles A, Moreno-Guerrero A, Parra-González María-Elena (2021). Projection of E-Learning in Higher Education: A Study of Its Scientific Production in Web of Science. Eur J Investig Health Psychol Educ.

[62] Fatima N, Abu KS (2019). E-learning Research Papers in Web of Science: A Biliometric Analysis. Library Philosophy and Practice.

[63] Tibaná-Herrera G, Fernández-Bajón M, De-Moya-Anegón F (2018). Output, collaboration and impact of e-learning research: Bibliometric analysis and visualizations at the country and institutional level (Scopus 2003-2016). EPI.

[64] Tibaná-Herrera G, Fernández-Bajón MT, De Moya-Anegón F (2018). Categorization of E-learning as an emerging discipline in the world publication system: a bibliometric study in SCOPUS. Int J Educ Technol High Educ.

[65] Tibaná-Herrera G, Fernández-Bajón MT, de Moya-Anegón F (2017). Global analysis of the E-learning scientific domain: a declining category?. Scientometrics.

[66] Sweileh WM (2021). Global Research Activity on E-Learning in Health Sciences Education: a Bibliometric Analysis. Med Sci Educ.

